# Melatonin Successfully Rescues the Hippocampal Molecular Machinery and Enhances Anti-oxidative Activity Following Early-Life Sleep Deprivation Injury

**DOI:** 10.3390/antiox10050774

**Published:** 2021-05-13

**Authors:** Hung-Ming Chang, Hsing-Chun Lin, Hsin-Lin Cheng, Chih-Kai Liao, To-Jung Tseng, Ting-Yi Renn, Chyn-Tair Lan, Li-You Chen

**Affiliations:** 1 Department of Anatomy and Cell Biology, School of Medicine, College of Medicine, Taipei Medical University, Taipei 11031, Taiwan; taiwanzoo@gmail.com (H.-M.C.); littlenorenn@gmail.com (T.-Y.R.); 2 Department of Nutrition, Chung Shan Medical University, Taichung 40201, Taiwan; cshc143@csh.org.tw (H.-C.L.); iamsamlee@livemail.tw (H.-L.C.); 3 Department of Nutrition, Chung Shan Medical University Hospital, Taichung 40201, Taiwan; 4 Department of Anatomy, School of Medicine, College of Medicine, Chung Shan Medical University, Taichung 40201, Taiwan; ckliiao37@csmu.edu.tw (C.-K.L.); tjtsenng@csmu.edu.tw (T.-J.T.); ctlan@csmu.edu.tw (C.-T.L.); 5 Department of Medical Education, Chung Shan Medical University Hospital, Taichung 40201, Taiwan

**Keywords:** early-life sleep deprivation, hippocampus, melatonin, neurochemical expression, TOF-SIMS analysis

## Abstract

Early-life sleep deprivation (ESD) is a serious condition with severe cognitive sequelae. Considering hippocampus plays an essential role in cognitive regulation, the present study aims to determine whether melatonin, a neuroendocrine beard with significant anti-oxidative activity, would greatly depress the hippocampal oxidative stress, improves the molecular machinery, and consequently exerts the neuro-protective effects following ESD. Male weanling Wistar rats (postnatal day 21) were subjected to ESD for three weeks. During this period, the animals were administered normal saline or melatonin (10 mg/kg) via intraperitoneal injection between 09:00 and 09:30 daily. After three cycles of ESD, the animals were kept under normal sleep/wake cycle until they reached adulthood and were sacrificed. The results indicated that ESD causes long-term effects, such as impairment of ionic distribution, interruption of the expressions of neurotransmitters and receptors, decreases in the levels of several antioxidant enzymes, and impairment of several signaling pathways, which contribute to neuronal death in hippocampal regions. Melatonin administration during ESD prevented these effects. Quantitative evaluation of cells also revealed a higher number of neurons in the melatonin-treated animals when compared with the saline-treated animals. As the hippocampus is critical to cognitive activity, preserving or even improving the hippocampal molecular machinery by melatonin during ESD not only helps us to better understand the underlying mechanisms of ESD-induced neuronal dysfunction, but also the therapeutic use of melatonin to counteract ESD-induced neuronal deficiency.

## 1. Introduction

It is well-known that a good night’s sleep revitalizes the body and mind, leading to better productivity and health [1,2]. However, with the advent of industrialization and popularization of consumer electronics, adults, children, and adolescents have suffered from sleep deprivation to differing extents. According to a report by the National Sleep Foundation of the US, increasing numbers of children are not experiencing sufficient sleep and nearly one-third suffer from chronic sleep disturbances [3]. Sleep deprivation can lead to a number of molecular, immune, and neural changes (such as changes in brain plasticity, ionic distribution, and gene expression) that cause a variety of neurobiological and psychological dysfunctions including behavioral and attention problems, impaired learning and memory, increased anxiety or depression, and perhaps emotional issues or cognitive dysfunction [4,5,6].

The hippocampus plays an essential role in regulating the cognitive function in which the neuronal processing during sleep is very important for mapping the recent memories to long-term storage [7]. The hippocampus contains several subfields (such as CA1, CA3 etc.) and each of them has a unique function in memory formation and consolidation [8]. It has been indicated that both the strongly recurrent collateral system of CA3 neurons and the largely parallel-organized CA1 neurons exhibit distinct local field potential signatures that participate in the modulation of memory consolidation during sleeping period [8]. Previous studies have reported that sleep deprivation would drastically decrease dendritic spine numbers of hippocampal CA1 neurons and reduces the connectivity between hippocampus and other brain regions [9,10]. Pharmacological reports have also demonstrated that impaired expression of neurotransmitters, such as serotonin (5-HT) and dopamine (DA), in limbic circuits causes cytoarchitectural changes in the dendritic spine that lead to cognitive dysfunction [11]. Pronounced synaptic transmission and sustained increase in synaptic efficiency, a molecular phenomenon known as long-term potentiation (LTP), plays an important role in learning and memory [12,13]. By activating *N*-methyl-d-aspartate receptors (NMDAR), enhanced calcium influx triggers intracellular signaling cascades involving a number of protein kinases, most importantly calcium/calmodulin kinase II (CaMKII), and drives the downstream cAMP response element binding protein (CREB) pathway [13]. Phosphorylation of CREB regulates transcription of the target genes responsible for the growth of dendritic spine, facilitating the maintenance of LTP and memory [14,15]. Through reciprocal augmentation of synaptic plasticity, the molecular machinery involved in LTP is of great importance in determining the integrity of emotional and cognitive functions. In a previous study, we demonstrated that decreased calcium intensity, together with depressed NMDAR-mediated neuronal nitric oxide synthase (nNOS) activation, positively correlates with poor performance in cognitive expression following total sleep deprivation [16]. As cognitive function is strongly modulated by LTP, preserving the spatio-temporal integration of related elements involved in hippocampal plasticity may lead to better emotional control and higher cognitive activity [13].

Oxidative stress is a harmful condition resulting from imbalance in generation and elimination of reactive oxygen species (ROS). Oxidative stress has been implicated in the pathophysiology of many neurological disorders [17,18]. Previous studies have indicated that sleep deprivation leads to oxidative stress that induces oxidative damage to lipids, resulting in alterations in membrane structures and reduction in intracellular antioxidant defense systems. Behavioral studies have also demonstrated that enhanced oxidative stress in the hippocampus causes cognitive deficits [19,20]. Excessive production of ROS and oxidative stress strengthen excitatory neurotransmission and depress inhibitory neurotransmission, which may lead to impairment of LTP and synaptic plasticity [21,22].

Sleep deprivation in early life can impose detrimental effects that persist throughout life [23]. Given that oxidative stress and impaired neuroplasticity serve as the major contributors to the pathogenesis of cognitive dysfunction, an effective anti-oxidative agent will be of great help in the clinical design of a therapeutic strategy to decrease neuronal dysfunction following early-life sleep deprivation (ESD).

Melatonin, the chief secretory product of the pineal gland, is best known for its effects on seasonal reproductive physiology, circadian rhythmicity, immune function, and anti-oxidative activity [24,25,26,27,28,29]. Over the past few years, there has been ample evidence to show that melatonin possesses neuro-protective functions [30,31,32]. Previous studies have demonstrated that melatonin improves cognitive dysfunction caused by total sleep deprivation, diabetes mellitus, pesticide exposure, and irradiation injury [33,34]. Melatonin also modulates NMDAR and serotonin release, enhances synaptic plasticity, and reduces oxidative stress in hippocampal region in experimental animal model [35,36]. 

Although destructive effects of sleep deprivation on neuronal function have been reported, the potential changes in neurochemical expression and the molecular mechanisms underlying the pathogenesis of neurological dysfunction induced by ESD have not. Moreover, the potential effects of melatonin on rescuing or preserving neuronal function following ESD remain to be explored. Therefore, the aims of the present study are to determine the possible long-term changes in the hippocampus and to further explore whether melatonin has potential protective effects on neuronal dysfunction and neurological damage following ESD.

## 2. Materials and Methods

### 2.1. Experimental Animals

Male weanling Wistar rats (n = 30, weighing 50–80 g each) were obtained from the National Laboratory Animal Center. They were randomly divided into three groups of 10 rats each. Rats in the first group were subjected to ESD for three cycles, with each cycle consisting of 5 days of total sleep deprivation followed by a 2-day break [23]. During the experimental sleep deprivation period, rats in this group were received 0.9% normal saline with 1% ethanol solution via intraperitoneal injection (ESD group). The second group was allowed to sleep (yoked control for sleep deprivation, ESC group). During the experimental sleep deprivation period, animals in the third group (ESD-M10) received 10 mg/kg melatonin (Sigma, St. Louis, MO, USA) via intraperitoneal injection [23]. As attempts to preclude the potential interference of endogenous melatonin, and to help better understand the distinct role of melatonin at specific dosage, melatonin was daily administrated between 09:00 and 09:30, a time point when the endogenous concentration of melatonin is at the lowest level. Melatonin was first dissolved in absolute alcohol, then diluted in normal saline with a final alcohol concentration of less than 1%. After the end of the three ESD cycles, all experimental animals were kept for 3 months until they reached adulthood. During this period, the rats were exposed to a 12:12 self-adjusting light-dark cycle (light from 07:00 to 19:00) at a constant temperature of 25 ± 1 °C and given ad libitum access to food and water. In the care and handling of all experimental animals, the Guide for the Care and Use of Laboratory Animals (1985), as stated in the US National Institutes of Health (NIH) guidelines (NIH publication No. 86-23), was followed. All drug administration procedures were approved by the Committee on the Care and Use of Laboratory Animals of Chung Shan Medical University (IACUC Approval No. 9687).

### 2.2. Sleep Deprivation Procedure

The previously described disc-on-water (DOW) method was applied [23,33,37,38]. An apparatus was used to induce sleep deprivation in experimental rats without excessive physical exertion. The yoked control group (ESC group) allowed to sleep although confined to the same apparatus, as the apparatus was turned off for a designated period of time [39]. Briefly, the apparatus was comprised of two rectangular clear plastic chambers (60 × 20 × 60 cm each) placed side by side. Beneath the disk floor were 5 cm deep trays that contained a small amount of water. Before the experiment, a rat to be sleep-deprived and its yoked control were placed in the apparatus for at least 3 days for environmental adaptation. During this period, the chambers were fitted with a solid mat in place of water. Sleep deprivation depended on rats’ hatred of water, as rats rarely enter water spontaneously. The disc was rotated slowly at a moderate speed of 3.5 rpm by computerized monitoring system. The rats had to keep pace to avoid stepping into the water. Previous studies have shown that the validated DOW setup causes experimental rats to be effectively deprived of sleep [38,39]. The animals were closely observed, including via camera surveillance, to ensure their active and safe movement on the platform or in the water. Food and water were provided ad libitum in the device and the physical condition and tolerance of the experimental rats were closely monitored. All sleep deprivation procedures were approved by the Laboratory Animal Center of Chung Shan Medical University.

### 2.3. Perfusion and Tissue Preparation

At the end of the experimental period (3 months after completion of ESD protocol), half of the animals in each group were subjected to transcardial perfusion for time-of-flight secondary ion mass spectrometry (TOF-SIMS) study and immunohistochemistry. First, animals were deeply anesthetized via intraperitoneal injection of ketamine (100 mg/kg) and xylazine (10–13 mg/kg) and subjected to transcardial perfusion with 300 mL of 0.9% normal saline followed by 4% paraformaldehyde in 0.1 M phosphate buffer (PB), pH 7.4. After perfusion, the hippocampus was removed and placed in the same fixative for 2 h. Tissue samples were immersed in graded concentrations of sucrose buffer (10~30%) for cryoprotection at 4 °C overnight. Serial 30 μm-thick sections of the hippocampus were cut transversely with a cryostat (CM3050 S, Leica Microsystems, Wetzlar, Germany) the next day.

For immunoblotting of related neurochemicals, another half of the animals in each group were deeply anesthetized via intraperitoneal injection of ketamine (100 mg/kg) and xylazine (10–13 mg/kg) and immediately decapitated at the end of their respective survival time points. After decapitation, the hippocampus was quickly removed and transferred to liquid nitrogen for later use. 

### 2.4. Immunohistochemistry

Immunohistochemical labeling of related neurochemicals was carried out using well-established methods [16,23]. The tissue sections collected in wells were first washed in three changes of 0.01 M phosphate buffer saline (PBS), pH 7.4, and then placed in 0.01 M PBS containing 10% methanol and 3% hydrogen peroxide for 1 h to reduce the endogenous peroxidase activity. Following three rinses in PBS, sections were incubated in the blocking medium containing 0.1% Triton X-100, 3% normal goat serum, and 2% bovine serum albumin for 1 h to block nonspecific binding. After washing, sections in the wells were separately incubated in primary antibody such as anti-CREB (1:4000, 9197 Cell Signaling, Danvers, MA, USA), anti-pCREB (1:200, 9198 Cell Signaling, Danvers, MA, USA), anti-Na^+^/K^+^ ATPase (1:250, 06-520 Millipore, Temecula, CA, USA), anti-γ-aminobutyric acid (GABA) (1:1000, 20094 Immunostar, Linscott, CA, USA), or anti-superoxide dismutase (SOD1) (GTX 100659, 1:500 GeneTex Inc., Irvine, CA, USA) with blocking medium at 4 °C overnight. After incubation in the primary antibody, the sections were further incubated with the biotinylated secondary antibody (1:200; Vector Laboratories, Burlingame, CA, USA) at room temperature for 2 h. This was followed by standard avidin-biotin complex (ABC) procedure with diaminobenzidine as a substrate of peroxidase. All reacted sections were rapidly dehydrated with graded alcohol, which was cleared with xylene, followed by coverslipping with Permount.

### 2.5. Nissl Staining

The tissue sections were washed twice in 0.01 M PBS for 15 min each and incubated for 10–15 min at room temperature in a 0.5% cresyl violet staining solution containing a few drops of glacial acetic acid. After incubation, the sections were washed with distilled water and gradually dehydrated in ethanol (70%, 95%, and 100%). Following dehydration, the sections were placed in xylene, followed by coverslipping with non-fluorescence mounting medium.

### 2.6. Quantitative Image Analysis for Light Microscopic (LM) Study

Only cell profiles with clear focal plane outlines were included in the analysis. The optical densities (ODs) of immunohistochemical staining products in the tissue sections were obtained with Image-Pro Plus software (Media Cybernetics, Silver Spring, MD, USA). A digital camera, mounted on Zeiss microscope (Axioplane 2, Carl Zeiss MicroImaging GmbH, Hamburg, Germany), was used to image the sections at 50× magnification in bright field, which were displayed on a high-resolution monitor. All OD readings from cells in each section were combined and averaged to obtain the total OD (TOD) of each section. An average of ten random rectangles (rectangular area = 150 μm^2^) in the lumen of the blood vessel were used to measure the background staining (BOD) of each region. By subtracting BOD from TOD, the true OD for each part of the background correction was obtained. For uniform settings, all images were captured on the same day. All parameters were carefully adjusted according to recommended gray levels, histogram stretching, and minimum OD. The Nissl straining was observed using a Zeiss microscope (Axioplane 2, Carl Zeiss MicroImaging GmbH, Hamburg, Germany), and these results were quantified using the Image J software (NIH, Bethesda, MD, USA).

### 2.7. Western Blotting

For processing the immunoblotting, the tissues removed from each experimental group were first collected in 0.01 M PBS with proteinase inhibitors [2 g/mL aprotinin, leupeptin, pepstatin A, and 120 g/mL phenylmethylsulfonyl fluoride (PMSF)], followed by homogenization (Polytron RT MR3100) for 3 × 10 s intervals. The homogenates were then spun at 13,200 rpm at 4 °C for 30 min to remove debris. Supernatants were aliquoted and the protein was assayed in 96-well plates by adding 3 μL of standard and 150 μL of protein assay dye reagent (Bio-Rad, Hercules, CA, USA) diluted 1:5 with distilled water. Absorbance at 595 nm was measured immediately on automated plate reader (Bio-Tek Instruments, Winooski, VT, USA). Equal amounts of protein were subjected to SDS gel electrophoresis and transferred to nylon membranes in an electrophoretic transfer cell (Trans-blot; BioRad). Immuno-detection was applied with antibodies specific to Na^+^/K^+^ ATPase (1:250, 06-520 Millipore, Temecula, CA, USA), serotonin receptor1 A (5-HT1 A) (1:500, AB15350 Millipore, Temecula, CA, USA), NMDA receptor (NMDAR) (1:1000, 2003687 Millipore, Temecula, CA, USA), SOD1 (GTX 100659, 1:1000, 3389944 Millipore, Temecula, CA, USA), catalase (CAT) (GTX 110704, 1:500 GeneTex Inc., Irvine, CA, USA), glutathione peroxidase (GSH-Px) (GTX 116040, 1:500 GeneTex Inc., Irvine, CA, USA), nuclear factor erythroid-2-related factor 2 (Nrf2) (AF0639, 1:1000, Affinity Biosciences, Cincinnati, OH, USA), phospho-Nrf2 (p-Nrf2) (1:300, Abcam, Cambridge, MA, USA), and rabbit anti-β-actin (1:5000, Bioworld Technology, St. Louis Park, MN, USA), respectively. Subsequent detection of the immuno-signal was achieved using the appropriate horseradish peroxidase (HRP)-conjugated secondary antibodies (1:5000, Sigma) at room temperature for 1 h and the reaction bands were visualized by chemiluminescence method (Renaissance kit; NEN, Boston, MA, USA). Signal intensity was quantified using ImageJ image analysis software (Version 1.4, NIH, Bethesda, MD, USA). Equal loading of proteins was confirmed by probing gels run in parallel with the housekeeping gene, β-actin. All OD readings were normalized for β-actin and expressed as mean ± standard deviation (SD).

### 2.8. TOF-SIMS Analysis

The in vivo ion expression was assessed on TOF-SIMS analysis using TOF-SIMS IV instrument (ION-TOF GmbH, Münster, Germany). Hippocampal tissue sections from each group were attached to silica wafer (1 cm × 1 cm), with the temperature of the sample holder adjusted to −60 °C. In this study, a gallium (Ga^+^) ion gun, operated at a voltage of 25 kV, was the main ion source (1 pA pulsed current). Ga^+^ primary ion beam was used to scan an area of 50 μm^2^ that included 62 × 62 pixels. Four different regions within the hippocampus were selected for scanning and four spectra were acquired from each sample. The image data acquisition time was 200 s and a pulse overflow gun with low-energy electrons was used for charge compensation. Major chamber vacuum was maintained between 10^−7^ and 10^−8^ Torr. The best resolution was m/Δm = 7450. Detection of positive and secondary ions flying through a reflection electron mass spectrometer was accomplished with a micro-channel plate assembly operated at 10 kV post-acceleration. Since the hippocampal slices were completely fixed in a large amount of paraformaldehyde, the paraformaldehyde molecules may have become the main element in the tissue matrix. Paraformaldehyde was used for mass calibration with a set of standard peaks, such as *m*/*z* 15 (CH3^+^), 27 (C2 H3^+^), 41 (C3 H5^+^), and 69 (Ga^+^), to improve the ion spectrum potential matrix effects. The positive ion SIMS spectrum of the molecule was controlled by strong fragments of *m*/*z* 23 and *m*/*z* 39 corresponding to sodium and potassium ions, respectively.

### 2.9. Statistical Analysis

All quantitative data obtained from the spectrometric and morphological studies of ESC, ESD and ESD-M10 groups were analyzed by one-way ANOVA followed by Bonferroni post hoc ANOVA test. *p* < 0.05 was considered statistically significant. 

## 3. Results

### 3.1. Melatonin Preserves Hippocampus Ion Homeostasis

**Figure 1:**
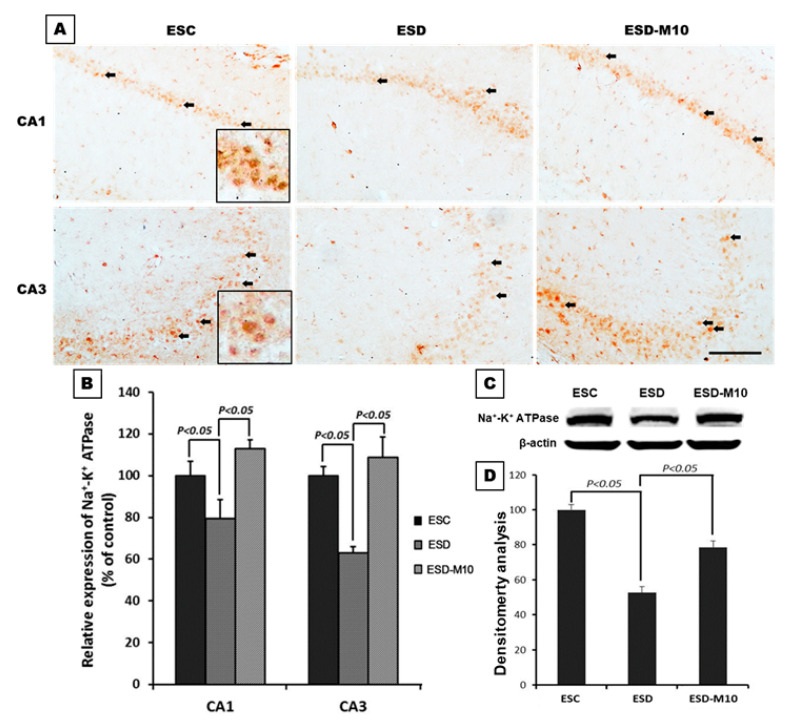
The effects of melatonin on Na^+^/K^+^ ATPase function. Photomicrographs (**A**) and histogram (**B**) show the extent of Na^+^/K^+^ ATPase expressions (arrow) in the CA1 and CA3 regions of hippocampus. Note that in ESC group, numerous neurons with strong Na^+^/K^+^ ATPase expressions were detected in the hippocampus. However, following ESD, remarkable decrease in hippocampal Na^+^/K^+^ ATPase expression was detected. In ESD-M10 group, hippocampal Na^+^/K^+^ ATPase expressions were effectively preserved. The immunoblotting data (**C**,**D**) coincided well with IHC findings in that melatonin effectively improved Na^+^/K^+^ ATPase activity. Scale bar: 300 μm.

**Figure 2:**
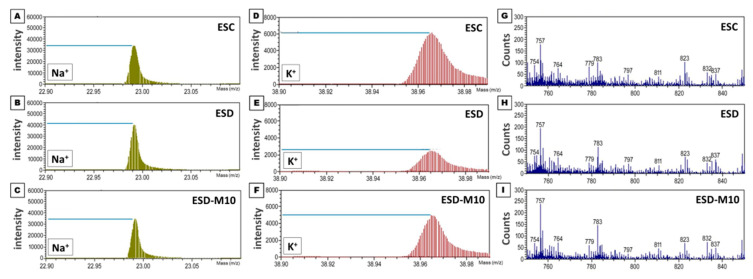
Melatonin restores the trans-membrane ionic gradient following ESD injury. TOF-SIMS positive spectra show the hippocampal Na^+^ and K^+^ expressions in the ESC group (**A**,**D**), ESD group (**B**,**E**), and ESD-M10 group (**C**,**F**). Note that in ESD rats, increases in hippocampal Na^+^ expression were more significant than in ESC and ESD-M10 rats (*p* < 0.05), but K^+^ expression was significantly reduced when compared with the other two groups (*p* < 0.05). Paraformaldehyde was used for mass calibration with a set of standard peaks to improve the effect of ion spectra on the substrate (**G**–**I**).

**Figure 3:**
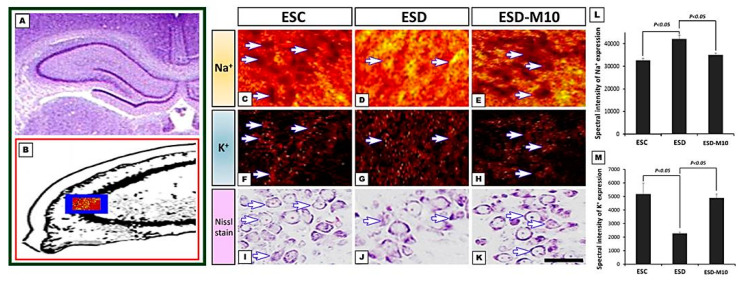
Photomicrograph (**A**), schematic diagram (**B**), positive ion images (**C**–**K**), and histograms (**L**,**M**) showed both Na^+^ and K^+^ expression in the hippocampus of ESC (**C**,**F**,**I**), ESD (**D**,**G**,**J**), and ESD with melatonin treatment (**E**,**H**,**K**) groups. Note that in ESC group, most of the hippocampal neurons (arrows in **C**) were devoid of Na^+^ signal in the intracellular portions. On the contrary, intense K^+^ signal was kept in the intracellular part of hippocampal neurons (arrows in **F**). However, following ESD, strong Na^+^ signal was accumulated in the intracellular portion of some hippocampal neurons (arrows in **D**), suggesting an impairment of Na^+^ pump was resulted from ESD. Nevertheless, after ESD and treated with melatonin, the distribution pattern of both Na^+^ and K^+^ was restored to nearly normal profile in which no significant Na^+^ signal was detected in the intracellular portion of hippocampal neurons (arrows in **E**). Quantitative data of the spectral intensity corresponded well the imaging findings (**L**,**M**). The blue box in (**B**) showed the detecting region of the ionic image. Scale bars: 50 μm.

The results of immunohistochemical staining of CA1 and CA3 regions of the hippocampus showed significant decreases in the neurons marked by Na^+^/K^+^ ATPase (indicated by arrows) in the ESD group when compared with the ESC group. However, in the ESD-M10 group, there was an increase in the neurons marked by Na^+^/K^+^ ATPase in the hippocampus (Figure 1A,B). On Western blot analysis, Na^+^/K^+^ ATPase protein expression in hippocampus was higher in ESC and ESD-M10 groups than in ESD group (Figure 1C,D). TOF-SIMS ion mass spectra data demonstrated the performance of sodium and potassium ions in the hippocampus. In Figure 2G–I, mass spectrum of paraformaldehyde served as the internal control. The positive ion spectrum showed that the intensity of the main peak of Na^+^ in the hippocampus of ESC rats is 32,620 ± 967.2 (Figure 2A). The Na^+^ intensity of the hippocampus in the ESD group was 42,000 ± 1632.93 (Figure 2B), which was significantly higher than that in the ESC group. However, the Na^+^ intensity of the melatonin-treated group (ESD-M10) was 35,000 ± 816.49 (Figure 2C), which was significantly lower than that of the ESD group. Changes in expression of K^+^ were in opposing directions to those of Na^+^. The mass spectrometry data indicated that the intensity of the K^+^ spectrum in the hippocampus of the ESD group (2280 ± 88.32) was significantly lower than that of the ESC (5166 ± 824.36) and ESD-M10 (4900 ± 294.39) groups (Figure 2D–F). The positive ion images showed that in ESC group, most of the hippocampal neurons were devoid of Na^+^ signal in the intracellular portions (Figure 3C). On the contrary, intense K^+^ signal was kept in the intracellular part of hippocampal neurons (Figure 3F). However, following ESD, strong Na^+^ signal was accumulated in the intracellular portion of some hippocampal neurons (Figure 3D). Nevertheless, after ESD and treated with melatonin, the distribution pattern of both Na^+^ and K^+^ was restored to nearly normal profile in which no significant Na^+^ signal was detected in the intracellular portion of hippocampal neurons (Figure 3E,H). Quantitative data corresponded well with the ionic imaging findings in which the intensity of Na^+^ was significantly higher in the ESD group (Figure 3L). Melatonin treatment successfully restored the ionic regulation to normal stage (Figure 3L,M). This suggests that impaired Na^+^/K^+^ ATPase activity causes accumulation of unexpectedly high levels of Na^+^ and low levels of K^+^ expression in the hippocampal neurons, resulting in abnormal ionic machinery in the hippocampus.

### 3.2. Melatonin Effectively Enhances the Expressions of GABA, NMDAR, and 5-HT1 A in the Hippocampus

**Figure 4:**
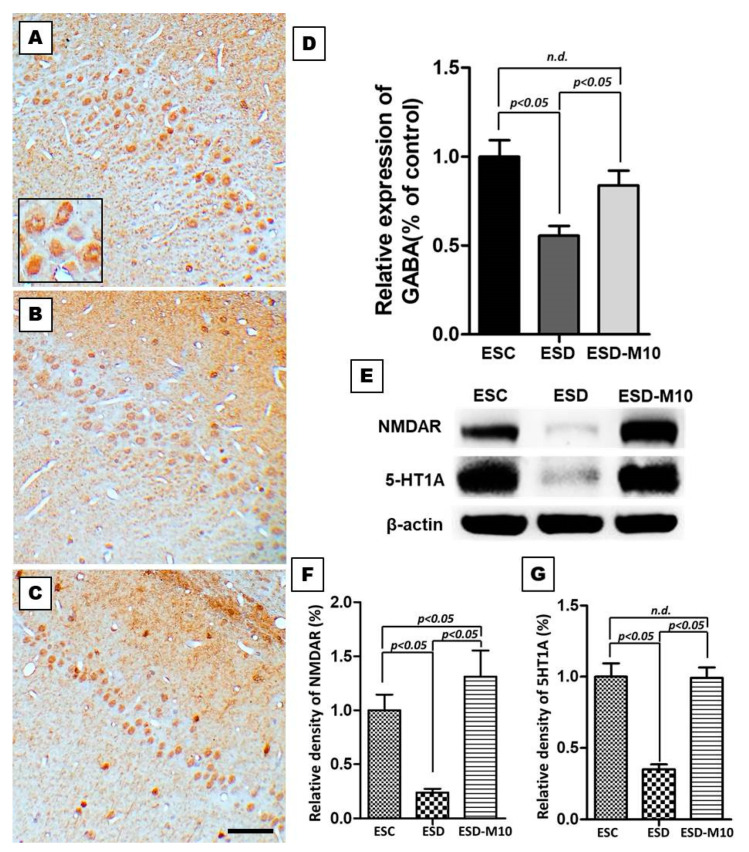
Photomicrographs (**A**–**C**) and histogram (**D**) show the extent of GABA expressions in the CA1 region of the hippocampus (**A**–**C**). Note that ESD rats demonstrated decreased GABAergic interneuron density in the hippocampus. In ESD-M10 group, the hippocampal GABA immune-expression significantly increased (**C**). Immunoblots (**E**) and histograms (**F**,**G**) show the protein expression level of 5-HT1 A and NMDAR in the hippocampus of ESC, ESD, and ESD-M10 groups. These activities were highest among ESC rats. Following ESD, protein expressions involved in neurotransmitter and receptor activities were significantly reduced (*p* < 0.05). However, in the animals treated with melatonin during the ESD period, effective expressions of 5-HT1 A and NMDAR significantly increased when compared with the ESD group (*p* < 0.05). Scale bars: 200 μm.

In the ESC group, GABA-immunoreactivity was detected in the CA1 region of the hippocampus (Figure 4A). In rats subjected to ESD, GABA-immunoreactivity in the hippocampus markedly decreased (Figure 4B). However, following melatonin treatment, GABA-immunoreactivity markedly increased (Figure 4C). Quantitative analysis revealed that the OD of hippocampal GABA staining is significantly reduced in ESD group when compared with ESC and ESD-M10 groups (Figure 4D). In addition we examined the effects of ESD on 5-HT1 A and NMDAR expressions in the hippocampus by Western blot. NMDAR and 5-HT1 A expressions were lower in ESD rats when compared with ESC rats. However, NMDAR and 5-HT1 A expressions increased significantly in animals treated with 10 mg/kg melatonin (ESD-M10 group) (Figure 4E–G).

### 3.3. Melatonin Increases p-CREB Protein Levels in Hippocampal Neurons in ESD Rats

**Figure 5:**
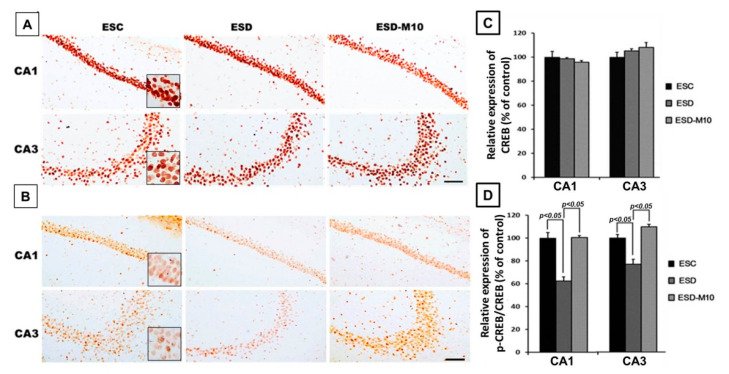
Photomicrographs (**A**,**B**) and histogram (**C**,**D**) show the extent of CREB and p-CREB activities in the CA1 and CA3 region of the hippocampus. Note that the protein expression levels of p-CREB markedly increased in the ESC and ESD-M10 groups when compared with the ESD group (*p* < 0.05). There was no difference between the three groups in terms of CREB protein expression in the hippocampus. Scale bars: 300 μm.

Activated NMDAR stimulates neuronal gene expression through the CREB signaling pathway [40]. CREB is a polymorphic transcription factor involved in cell survival, neuronal plasticity, addiction, neurogenesis, learning, and memory. Therefore, we used immunohistochemical staining to measure and performance with expression levels of CREB in the hippocampus. The results showed no significant differences in CREB expression in the hippocampus among the three groups (Figure 5A,C). However, in animals subjected to ESD without melatonin, p-CREB expression drastically decreased in the hippocampus when compared with the other two groups (Figure 5B). Quantitative data showed that the p-CREB/CREB ratio of the ESD group is significantly lower than that of the ESC and ESD-M10 groups (Figure 5D). The decrement of hippocampal NMDAR and 5-HT1 A corresponded well with reduced p-CREB immunoreactivity. These results suggested that ESD leads to long-term neurochemical imbalances in the hippocampus. However, melatonin ameliorates this damage.

### 3.4. Melatonin Effectively Promotes p-Nrf2, Anti-oxidative Enzyme Activities, and Neuronal Cell Survival in the Hippocampus

**Figure 6:**
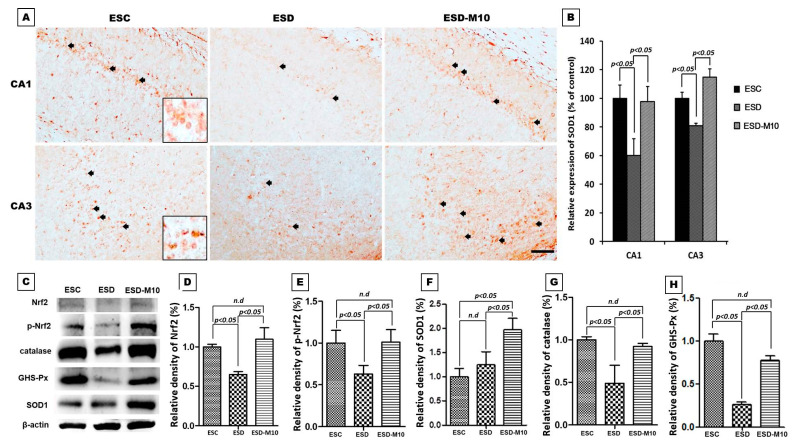
Photomicrographs (**A**) and histogram (**B**) show SOD1 expressions in the CA1 and CA3 region of the hippocampus based on immunohistochemistry results. Note that ESD rats showed decreased SOD1 expression in the hippocampus. In the ESC and ESD-M10 groups, hippocampal scheme 1 immune-expression significantly increased (*p* < 0.05). Immunoblots (**C**) and histograms (**D**–**H**) show the expressions of Nrf2 (D), p-Nrf2 (**E**) and the downstream antioxidant enzymes including SOD1 (**F**), CAT (**G**), and GSH-Px (**H**) of ESD-M10 group which were higher than in ESD group (*p* < 0.05). Note that melatonin effectively promotes anti-oxidative enzyme activities. Scale bars: 300 μm.

Melatonin is an antioxidant that has been proven effective in reducing oxidative damage to the central nervous system. Melatonin stimulates Nrf2 expression, thereby increasing the activity of downstream antioxidant enzymes including SOD1, glutathione, and peroxidase [24,41,42]. Therefore, we used immunohistochemical and immunoblotting analyses to investigate whether treatment with melatonin during ESD activates the antioxidant enzymes of the hippocampus and Nissl staining to explore the functional activity of neuronal cells. The results of immunohistochemical staining showed that SOD1 is significantly reduced in the ESD group compared to the ESC group. In the ESD-M10 group, an increase in the number of cells labeled with SOD1 was observed (Figure 6A,B). The results of immunoblotting also showed that after melatonin treatment, the activities of Nrf2, p-Nrf2 and expressions of antioxidant enzymes in the hippocampus (i.e., SOD1, catalase and GSH-Px) were all significantly increased (Figure 6C–H). 

**Figure 7:**
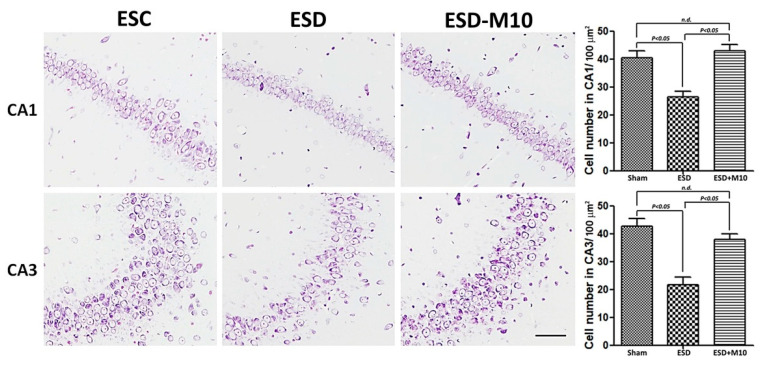
Photomicrographs and histogram show the results of Nissl staining of hippocampal neurons. There were decreases in cell density in CA1 and CA3 following ESD. Staining intensity increased with melatonin treatment. The histogram shows the quantification of the hippocampal CA1 and CA3 neurons. Nissl stained hippocampal neurons significantly increased in ESC and ESD-M10 groups when compared with ESD group (*p* < 0.05). Scale bars: 100 μm.

Based on the results of immunohistochemistry, immunoblotting, and TOF-SIMS analyses, ESD leads to long-term imbalances in the distributions of ions (Figure 1, Figure 2 and Figure 3), abnormalities in neurotransmitters and receptors (Figure 4 and Figure 5), and decreases in antioxidant enzymes (Figure 6). Based on the results of Nissl staining (Figure 7), intact neurons in the hippocampus of ESD group rats were markedly reduced. The intact neuron numbers per 100 mm^2^ in the ESC group were higher than in the ESD-M10 group, but the difference was not significant.

## 4. Discussion

The present study provides morphological evidence that sleep deprivation in early life not only leads to short-term effects but also to long-term damage. In addition, this study provides functional anatomical evidence that melatonin treatment effectively rescues neuronal abnormalities in the hippocampus of adult rats after ESD. Early-life stress is a risk factor for the persistence and development of mental disorders [43,44]. ESD is a major stressor in children. Epidemiological and psychological studies have indicated that sleep insufficiency during childhood induces vulnerability to the effects of stress later in life, in which the persistent changes in neuronal plasticity in the hippocampus represent the underlying neuronal substrate for the development of anxiety, depression, and cognitive deficiencies [45,46,47]. Our previous report has shown that chronic ESD significantly impairs the pineal signaling and biosynthesis of melatonin, suggesting that distress experienced in very early-life (i.e., in weanlings) not only causes short-term consequences, but also lifelong detrimental effects [23]. It is noteworthy that the relationship between ESD and the development of major mental disorders has drawn much attention recently. In this study, imbalances in the distributions of ions (Figure 1, Figure 2 and Figure 3), abnormalities in neurotransmitters and receptors (Figure 4 and Figure 5), decreases in antioxidant enzymes (Figure 6), and loss of functional activity of neuronal cells (Figure 7) were observed in ESD rats. These deleterious effects of ESD on the nervous system persisted into adulthood, as changes in the hippocampus were detected 3 months following the termination of ESD.

Na^+^/K^+^ ATPase is critical for regulating intracellular pH, cell volume, and calcium concentration, as well as for exchanging solutes transferred through coupled systems. The transmembrane Na^+^ gradient mainly flows from Na^+^ through the sodium channel and is discharged through the activity of Na^+^/K^+^ ATPase. Previous research has indicated that the inhibition of Na^+^/K^+^ ATPase activity in the brain causes central nervous system edema and cell death and interferes with learning and memory processes [48]. Inactivation of Na^+^/K^+^ ATPase reduces the membrane barrier to Na^+^ movement, making it easier for it to enter the cytoplasm [49]. This was the case in our current study, as excessive Na^+^ influx was detected in the hippocampus (Figure 2 and Figure 3) due to reduced Na^+^/K^+^ ATPase activity in the ESD group (Figure 1). As a result, excess sodium ions accumulate in cells and alter Na^+^/K^+^ transferase or other ion transfer systems, causing cell swelling and damage [50]. Significant decrease in Na^+^/K^+^ ATPase density is observed in patients with psychiatric disorders [51]. Moreover, Na^+^/K^+^ ATPase regulates and affects neurotransmitters and receptors such as GABA, 5-HT1 A, and NMDAR [52]. In the present study, GABA-immunoreactivity neurons significantly decreased in ESD group (Figure 4A–D). Glutamate is involved in brain metabolism and acts as a precursor to different compounds such as the inhibitory neurotransmitter GABA. GABAergic neurons are distributed throughout the central nervous system and provide inhibitory control of projection neurons. Abnormal GABAergic neurotransmission has been shown to cause various neuropsychiatric diseases [53,54]. However, chronic sleep deprivation in early life also causes long-term changes in neurotransmitters and receptors in the hippocampus. Activation of NMDAR alters intracellular Na^+^ and K^+^ concentrations, which are subsequently restored by Na^+^/K^+^ ATPase. Na^+^/K^+^ ATPase and NMDAR functionally interact by forming macromolecular complexes to restore ionic homeostasis after the excitation of the neurons [55]. Our immunoblot data indicated that 5-HT1 A and NMDAR expressions in the hippocampus are notably higher in the ESC group (Figure 4E–G). NMDAR is understood to play a key role in synaptic plasticity and memory function. Abnormalities in the NMDAR pathway can lead to complex psychiatric disorders with multiple symptoms, including psychosis [56]. However, serotonin has been shown to increase cellular excitability by several mechanisms and previous studies have demonstrated the ability of 5-HT1 Ato modulate NMDAR activity and facilitate LTP expression in CA1 [57,58]. By activating NMDAR, Ca^2+^ flows into cells, triggering a signal transduction response that drives the CREB pathway, thereby promoting LTP, synaptic plasticity, and memory maintenance [13,14,15]. In addition, NMDAR promotes developmental regulatory switches in intracellular signaling pathways that may contribute to neuroplasticity in the developing brain. During the maturation of neurons, NMDAR stimulates the gene expressions of neurons through Ser-133 of CREB protein phosphatase pathway [59]. From our experimental results, the expression of p-CREB in the hippocampus decreased following ESD (Figure 5). 

Furthermore, ESD reduced the activity of nuclear factor erythroid 2-related factor 2 and antioxidant responsive element (Nrf2-ARE) in hippocampal neurons, as well as the activities of SOD1, CAT, and GSH-Px (Figure 6). These findings illustrated that hippocampal neuronal cells are susceptible to oxidative damage and that oxidative stress plays a pivotal role in cytotoxicity induced by sleep deprivation. Numerous reports have suggested that sleep deprivation triggers increased oxidative stress in the brain, causing damage to neuronal cells, which in turn leads to cognitive and memory dysfunction [60,61]. Na^+^/K^+^ ATPase is highly sensitive to oxidative damage and free radical attack [62]. The interruption of CREB signal transduction may be due to a disease-dependent increase in oxidative load. It has been suggested that CREB acts as a key upstream integrator of neuroprotective signaling against ROS-mediated cell death [63]. In this study, ESD led to imbalances in neuronal cell ion distribution, neurotransmitters, receptors, associated downstream proteins, and antioxidant factors, possibly resulting in neuronal cell death (Figure 7). We speculate that the stress of sleep deprivation in early childhood results in LTP impairment, neuroplasticity dysfunction, and memory deficit in adulthood. In this study, we demonstrated that ESD causes long-term alterations in the hippocampus, which may be the key factor in intracellular and extracellular ion imbalances.

In our previous study, we demonstrated that ESD not only affects melatonin secretion, but also leads to metabolic dysfunction in adulthood [23]. Melatonin is a secreted product of the pineal body that is involved in the regulation of circadian rhythms and many physiological functions, as well as has neuroprotective effects. It also acts as an effective antioxidant and a powerful free radical scavenger [64,65]. The efficacy of melatonin in scavenging various free radicals may result from its ability to stimulate antioxidant enzymes [66]. Melatonin activates Nrf2-ARE signaling pathway, thereby promoting the activity of downstream antioxidant enzymes, including SOD1, glutathione, and peroxidase, and further protecting neurons [67]. In this study, we also clearly demonstrated that after sleep deprivation, melatonin effectively promotes p-Nrf2 expression and increases the activities of downstream antioxidant enzymes (i.e., SOD, CAT, and GSH-Px) (Figure 6). Furthermore, melatonin increases and normalizes Na^+^/K^+^ ATPase activity [68,69]. In the present study, melatonin altered Na^+^/K^+^ ATPase activity (Figure 1) and progressively affected cellular ion distribution, neuro-receptors, downstream signaling proteins, and antioxidant enzyme properties (Figure 2, Figure 3, Figure 4, Figure 5 and Figure 6), as well as improved neuronal cell survival (Figure 7) in hippocampus following ESD. Based on morphological results, experimental animals receiving melatonin showed significant improvement in the neurological imbalances induced by sleep deprivation.

## 5. Conclusions

**Figure 8:**
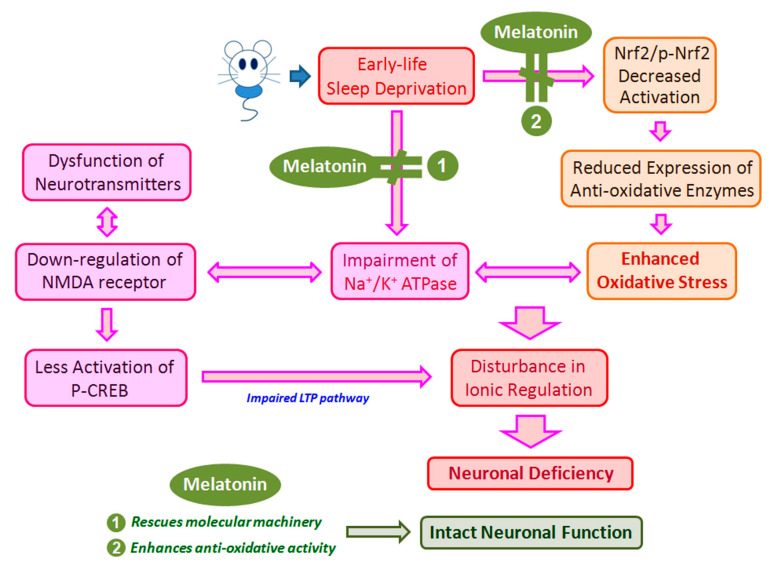
Schematic diagram showed the potential mechanism(s) of early-life sleep deprivation (ESD) on the induction or development of neuronal deficiency. ESD would both impair the Na^+^/K^+^ ATPase and depress the Nrf2-mediated anti-oxidative enzymes activities that consequently lead to neuronal deficiency through enhanced oxidative stress and cellular bioenergetics disruption. Exogenous application of melatonin could successfully preserve the neuronal function through effectively rescuing the molecular machinery of the neurons and significantly increasing neuronal anti-oxidative activity.

Adequate sleep is essential for the normal functioning of the brain, especially in the early stages of life, as after birth there is a critical period of maturation of the brain. Based on these findings, this study provides evidence that chronic ESD severely impairs hippocampal signaling and antioxidant enzymes and causes neurological dysfunction in adulthood (Figure 8). Tremendous stress in the early stages of life not only has short-term consequences, but also long-term consequences. Melatonin has the potential to protect against cognitive abnormalities and neurological dysfunctions caused by sleep deprivation in early life (Figure 8). Thus, the results of this study not only increase our understanding of the pathogenesis of ESD-induced neuronal deficiency, but also extend our current knowledge of the therapeutic use of melatonin in forestalling neuronal dysfunction induced by ESD.

## Data Availability

Not applicable.

## Abbreviations

CREB	cAMP response element binding protein
ESD	early-life sleep deprivation
GABA	γ-aminobutyric acid
GSH-Px	glutathione peroxidase
LTP	long-term potentiation
NMDA	*N*-methyl-d-aspartate
Nrf2	nuclear factor E2-related factor 2
ROS	reactive oxygen species
SOD1	superoxide dismutase
TOF-SIMS	time-of-flight secondary ion mass spectrometry

## References

[1] Palagini L., Rosenlicht N. (2011). Sleep, dreaming, and mental health: A review of historical and neurobiological perspectives. Sleep Med. Rev..

[2] Grandner M.A. (2020). Sleep, health, and society. Sleep Med. Clin..

[3] (2004). Sleep in America Poll-Children and Sleep. National Sleep Fundation.

[4] Banks S., Dinges D.F. (2007). Behavioral and physiological consequences of sleep restriction. J. Clin. Sleep Med..

[5] Ford D.E., Kamerow D.B. (1989). Epidemiologic study of sleep disturbances and psychiatric disorders. An opportunity for prevention?. JAMA.

[6] Walker M.P. (2008). Cognitive consequences of sleep and sleep loss. Sleep Med..

[7] Sawangjit A., Oyanedel C.N., Niethard N., Salazar C., Born J., Inostroza M. (2018). The hippocampus is crucial for forming non-hippocampal long-term memory during Sleep. Nature.

[8] Malerba P., Bazhenov M. (2019). Circuit mechanisms of hippocampal reactivation during Sleep. Neurobiol. Learn. Mem..

[9] Havekes R., Vecsey C.G., Abel T. (2012). The impact of sleep deprivation on neuronal and glial signaling pathways important for memory and synaptic plasticity. Cell. Signal..

[10] Havekes R., Park A.J., Tudor J.C., Luczak V.G., Hansen R.T., Ferri S.L., Bruinenberg V.M., Poplawski S.G., Day J.P., Aton S.J. (2016). Sleep deprivation causes memory deficits by negatively impacting neuronal connectivity in hippocampal area CA1. Elife.

[11] Gonzalez-Burgos I., Feria-Velasco A. (2008). Serotonin/dopamine interaction in memory formation. Prog. Brain Res..

[12] Martinez J.L., Derrick B.E. (1996). Long-term potentiation and learning. Annu. Rev. Psychol..

[13] Lynch M.A. (2004). Long-term potentiation and memory. Physiol. Rev..

[14] Murphy D.D., Segal M. (1997). Morphological plasticity of dendritic spines in central neurons is mediated by activation of cAMP response element binding protein. Proc. Natl. Acad. Sci. USA.

[15] Miyamoto E. (2006). Molecular mechanism of neuronal plasticity: Induction and maintenance of long-term potentiation in the hippocampus. J. Pharmacol. Sci..

[16] Chang H.M., Liao W.C., Sheu J.N., Chang C.C., Lan C.T., Mai F.D. (2012). Sleep deprivation impairs Ca^2+^ expression in the hippocampus: Ionic imaging analysis for cognitive deficiency with TOF-SIMS. Microsc. Microanal..

[17] Reynolds A., Laurie C., Mosley R.L., Gendelman H.E. (2007). Oxidative stress and the pathogenesis of neurodegenerative disorders. Int. Rev. Neurobiol..

[18] Li J., O W., Li W., Jiang Z.G., Ghanbari H.A. (2013). Oxidative stress and neurodegenerative disorders. Int. J. Mol. Sci..

[19] Silva R.H., Abilio V.C., Takatsu A.L., Kameda S.R., Grassl C., Chehin A.B., Medrano W.A., Calzavara M.B., Registro S., Andersen M.L. (2004). Role of hippocampal oxidative stress in memory deficits induced by sleep deprivation in mice. Neuropharmacology.

[20] D’Almeida V., Lobo L.L., Hipolide D.C., de Oliveira A.C., Nobrega J.N., Tufik S. (1998). Sleep deprivation induces brain region-specific decreases in glutathione levels. Neuroreport.

[21] Wu L.J., Kim S.S., Zhuo M. (2008). Molecular targets of anxiety: From membrane to nucleus. Neurochem. Res..

[22] Davis M., Rainnie D., Cassell M. (1994). Neurotransmission in the rat amygdala related to fear and anxiety. Trends Neurosci..

[23] Chen L.Y., Tiong C., Tsai C.H., Liao W.C., Yang S.F., Youn S.C., Mai F.D., Chang H.M. (2015). Early-life sleep deprivation persistently depresses melatonin production and bio-energetics of the pineal gland: Potential implications for the development of metabolic deficiency. Brain Struct. Funct..

[24] Garcia J.J., Lopez-Pingarron L., Almeida-Souza P., Tres A., Escudero P., Garcia-Gil F.A., Tan D.X., Reiter R.J., Ramirez J.M., Bernal-Perez M. (2014). Protective effects of melatonin in reducing oxidative stress and in preserving the fluidity of biological membranes: A review. J. Pineal Res..

[25] Litvinenko G.I., Shurlygina A.V., Gritsyk O.B., Mel’nikova E.V., Tenditnik M.V., Avrorov P.A., Trufakin V.A. (2015). Effects of melatonin on morphological and functional parameters of the pineal gland and organs of immune system in rats during natural light cycle and constant illumination. Bull. Exp. Biol. Med..

[26] Reiter R.J., Mayo J.C., Tan D.X., Sainz R.M., Alatorre-Jimenez M., Qin L. (2016). Melatonin as an antioxidant: Under promises but over delivers. J. Pineal Res..

[27] Favero G., Bonomini F., Franco C., Rezzani R. (2019). Mitochondrial dysfunction in skeletal muscle of a fibromyalgia model: The potential benefits of melatonin. Int. J. Mol. Sci..

[28] Tan D.X., Hardeland R. (2020). Targeting host defense system and rescuing compromised mitochondria to increase tolerance against pathogens by melatonin may impact outcome of deadly virus infection pertinent to COVID-19. Molecules.

[29] Fernández-Palanca P., Méndez-Blanco C., Fondevila F., Tuñón M.J., Reiter R.J., Mauriz J.L., González-Gallego J. (2021). Melatonin as an antitumor agent against liver cancer: An updated systematic review. Antioxidants.

[30] Antolin I., Rodriguez C., Sainz R.M., Mayo J.C., Uria H., Kotler M.L., Rodriguez-Colunga M.J., Tolivia D., Menendez-Pelaez A. (1996). Neurohormone melatonin prevents cell damage: Effect on gene expression for antioxidant enzymes. FASEB J..

[31] Tan D.X., Manchester L.C., Terron M.P., Flores L.J., Reiter R.J. (2007). One molecule, many derivatives: A never-ending interaction of melatonin with reactive oxygen and nitrogen species?. J. Pineal Res..

[32] Reiter R.J., Tan D.X., Jou M.J., Korkmaz A., Manchester L.C., Paredes S.D. (2008). Biogenic amines in the reduction of oxidative stress: Melatonin and its metabolites. Neuroendocrinol. Lett..

[33] Chang H.M., Wu U.I., Lan C.T. (2009). Melatonin preserves longevity protein (sirtuin 1) expression in the hippocampus of total sleep-deprived rats. J. Pineal Res..

[34] Banke I.S., Folorunsho A.S., Mohammed B., Musa S.M., Charles O., Olusegun A.J. (2014). Effects of melatonin on changes in cognitive performances and brain malondialdehyde concentration induced by sub-chronic co-administration of chlorpyrifos and cypermethrin in male Wister rats. Asian Pac. J. Trop. Biomed..

[35] Dilek M., Naziroglu M., Baha Oral H., Suat Ovey I., Kucukayaz M., Mungan M.T., Kara H.Y., Sutcu R. (2010). Melatonin modulates hippocampus NMDA receptors, blood and brain oxidative stress levels in ovariectomized rats. J. Membr. Biol..

[36] El-Sherif Y., Tesoriero J., Hogan M.V., Wieraszko A. (2003). Melatonin regulates neuronal plasticity in the hippocampus. J. Neurosci. Res..

[37] Everson C.A., Bergmann B.M., Rechtschaffen A. (1989). Sleep deprivation in the rat: III. Total sleep deprivation. Sleep.

[38] Rechtschaffen A., Bergmann B.M. (1995). Sleep deprivation in the rat by the disk-over-water method. Behav. Brain Res..

[39] Bergmann B.M., Kushida C.A., Everson C.A., Gilliland M.A., Obermeyer W., Rechtschaffen A. (1989). Sleep deprivation in the rat: II. Methodology. Sleep.

[40] Shaywitz A.J., Greenberg M.E. (1999). CREB: A stimulus-induced transcription factor activated by a diverse array of extracellular signals. Annu. Rev. Biochem..

[41] Rodriguez C., Mayo J.C., Sainz R.M., Antolin I., Herrera F., Martin V., Reiter R.J. (2004). Regulation of antioxidant enzymes: A significant role for melatonin. J. Pineal Res..

[42] Chen L.Y., Renn T.Y., Liao W.C., Mai F.D., Ho Y.J., Hsiao G., Lee A.W., Chang H.M. (2017). Melatonin successfully rescues hippocampal bioenergetics and improves cognitive function following drug intoxication by promoting Nrf2-ARE signaling activity. J. Pineal Res..

[43] Famularo R., Kinscherff R., Fenton T. (1992). Psychiatric diagnoses of maltreated children: Preliminary findings. J. Am. Acad. Child Adolesc. Psychiatry.

[44] Heim C., Nemeroff C.B. (2001). The role of childhood trauma in the neurobiology of mood and anxiety disorders: Preclinical and clinical studies. Biol. Psychiatry.

[45] Taylor D.J., Lichstein K.L., Durrence H.H., Reidel B.W., Bush A.J. (2005). Epidemiology of insomnia, depression, and anxiety. Sleep.

[46] Harvey A.G. (2009). The adverse consequences of sleep disturbance in pediatric bipolar disorder: Implications for intervention. Child Adolesc. Psychiatr. Clin. N. Am..

[47] Sterpenich V., Albouy G., Darsaud A., Schmidt C., Vandewalle G., Dang Vu T.T., Desseilles M., Phillips C., Degueldre C., Balteau E. (2009). Sleep promotes the neural reorganization of remote emotional memory. J. Neurosci..

[48] Dos Reis E.A., de Oliveira L.S., Lamers M.L., Netto C.A., Wyse A.T. (2002). Arginine administration inhibits hippocampal Na(+),K(+)-ATPase activity and impairs retention of an inhibitory avoidance task in rats. Brain Res..

[49] Amaral A.U., Seminotti B., Cecatto C., Fernandes C.G., Busanello E.N., Zanatta A., Kist L.W., Bogo M.R., de Souza D.O., Woontner M. (2012). Reduction of Na+, K+-ATPase activity and expression in cerebral cortex of glutaryl-CoA dehydrogenase deficient mice: A possible mechanism for brain injury in glutaric aciduria type I. Mol. Genet. Metab..

[50] Mandal J., Chakraborty A., Chandra A.K. (2016). Altered acetylcholinesterase and Na+-K+ATPase activities in different areas of brain in relation to thyroid gland function and morphology under the influence of excess iodine. Int. J. Pharm. Clin. Res..

[51] Goldstein I., Levy T., Galili D., Ovadia H., Yirmiya R., Rosen H., Lichtstein D. (2006). Involvement of Na(+), K(+)-ATPase and endogenous digitalis-like compounds in depressive disorders. Biol. Psychiatry.

[52] Pivovarov A.S., Calahorro F., Walker R.J. (2018). Na(+)/K(+)-pump and neurotransmitter membrane receptors. Invert. Neurosci..

[53] Tremblay R., Lee S., Rudy B. (2016). GABAergic interneurons in the neocortex: From cellular properties to circuits. Neuron.

[54] Taylor S.F., Tso I.F. (2015). GABA abnormalities in schizophrenia: A methodological review of in vivo studies. Schizophr. Res..

[55] Akkuratov E.E., Lopacheva O.M., Kruusmagi M., Lopachev A.V., Shah Z.A., Boldyrev A.A., Liu L. (2015). Functional interaction between Na/K-ATPase and NMDA receptor in cerebellar neurons. Mol. Neurobiol..

[56] Lakhan S.E., Caro M., Hadzimichalis N. (2013). NMDA receptor activity in neuropsychiatric disorders. Front. Psychiatry.

[57] Zhong P., Yuen E.Y., Yan Z. (2008). Modulation of neuronal excitability by serotonin-NMDA interactions in prefrontal cortex. Mol. Cell Neurosci..

[58] Vasefi M.S., Yang K., Li J., Kruk J.S., Heikkila J.J., Jackson M.F., MacDonald J.F., Beazely M.A. (2013). Acute 5-HT7 receptor activation increases NMDA-evoked currents and differentially alters NMDA receptor subunit phosphorylation and trafficking in hippocampal neurons. Mol. Brain.

[59] Sala C., Rudolph-Correia S., Sheng M. (2000). Developmentally regulated NMDA receptor-dependent dephosphorylation of cAMP response element-binding protein (CREB) in hippocampal neurons. J. Neurosci..

[60] Atrooz F., Salim S. (2020). Sleep deprivation, oxidative stress and inflammation. Adv. Protein Chem. Struct. Biol..

[61] Nabaee E., Kesmati M., Shahriari A., Khajehpour L., Torabi M. (2018). Cognitive and hippocampus biochemical changes following sleep deprivation in the adult male rat. Biomed. Pharmacother..

[62] Wang X.Q., Xiao A.Y., Sheline C., Hyrc K., Yang A., Goldberg M.P., Choi D.W., Yu S.P. (2003). Apoptotic insults impair Na^+^, K^+^-ATPase activity as a mechanism of neuronal death mediated by concurrent ATP deficiency and oxidant stress. J. Cell Sci..

[63] Lee B., Cao R., Choi Y.S., Cho H.Y., Rhee A.D., Hah C.K., Hoyt K.R., Obrietan K. (2009). The CREB/CRE transcriptional pathway: Protection against oxidative stress-mediated neuronal cell death. J. Neurochem..

[64] Reiter R.J., Acuna-Castroviejo D., Tan D.X., Burkhardt S. (2001). Free radical-mediated molecular damage. Mechanisms for the protective actions of melatonin in the central nervous system. Ann. N. Y. Acad. Sci..

[65] Reiter R.J., Tan D.X., Manchester L.C., Lopez-Burillo S., Sainz R.M., Mayo J.C. (2003). Melatonin: Detoxification of oxygen and nitrogen-based toxic reactants. Adv. Exp. Med. Biol..

[66] Gonzalez M.A., Contini Mdel C., Millen N., Mahieu S.T. (2012). Role of melatonin in the oxidative damage prevention at different times of hepatic regeneration. Cell Biochem. Funct..

[67] Wang Z., Ma C., Meng C.J., Zhu G.Q., Sun X.B., Huo L., Zhang J., Liu H.X., He W.C., Shen X.M. (2012). Melatonin activates the Nrf2-ARE pathway when it protects against early brain injury in a subarachnoid hemorrhage model. J. Pineal Res..

[68] Ritter L., Kleemann D., Hickmann F.H., Amaral A.U., Sitta A., Wajner M., Ribeiro C.A. (2015). Disturbance of energy and redox homeostasis and reduction of Na+,K+-ATPase activity provoked by in vivo intracerebral administration of ethylmalonic acid to young rats. Biochim. Biophys. Acta.

[69] Oliveira-Abreu K., Silva-Dos-Santos N.M., Coelho-de-Souza A.N., Ferreira-da-Silva F.W., Silva-Alves K.S.D., Cardoso-Teixeira A.C., Cipolla-Neto J., Leal-Cardoso J.H. (2019). Melatonin reduces excitability in dorsal root ganglia neurons with inflection on the repolarization phase of the action potential. Int. J. Mol. Sci..

